# Cerebrospinal fluid cytokines in metastatic group 3 and 4 medulloblastoma

**DOI:** 10.1186/s12885-020-07048-0

**Published:** 2020-06-15

**Authors:** Sharon Y. Y. Low, Nurfahanah Bte Syed Sulaiman, Enrica E. K. Tan, Lee Ping Ng, Chik Hong Kuick, Kenneth T. E. Chang, Phua Hwee Tang, Ru Xin Wong, Wen Shen Looi, David C. Y. Low, Wan Tew Seow

**Affiliations:** 1 Neurosurgical Service, KK Women’s and Children’s Hospital, 100 Bukit Timah Road, Singapore, 229899 Singapore; 2 Department of Neurosurgery, National Neuroscience Institute, 11 Jalan Tan Tock Seng, Singapore, 308433 Singapore; 3 SingHealth Duke-NUS Neuroscience Academic Clinical Program, 11 Jalan Tan Tock Seng, Singapore, 308433 Singapore; 4 VIVA-KKH Paediatric Brain and Solid Tumours Laboratory, 100 Bukit Timah Road, Singapore, 229899 Singapore; 5 Paediatric Haematology/Oncology Service, KK Women’s and Children’s Hospital, 100 Bukit Timah Road, Singapore, 229899 Singapore; 6 Department of Pathology and Laboratory Medicine, KK Women’s and Children’s Hospital, 100 Bukit Timah Road, Singapore, 229899 Singapore; 7 Department of Diagnostic and Interventional Imaging, KK Women’s and Children’s Hospital, 100 Bukit Timah Road, Singapore, 229899 Singapore; 8 Department of Radiation Oncology, 11 Hospital Drive, Singapore, 169610 Singapore

**Keywords:** Cerebrospinal fluid, Cytokines, Medulloblastoma, Metastasis

## Abstract

**Background:**

Metastatic medulloblastoma (MB) portends a poor prognosis. Amongst the 4 molecular subtypes, Group 3 and Group 4 patients have a higher incidence of metastatic disease, especially involving the neuroaxis. At present, mechanisms underlying MB metastasis remain elusive. Separately, inflammation has been implicated as a key player in tumour development and metastasis. Cytokines and their inflammation-related partners have been demonstrated to act on autocrine and, or paracrine pathways within the tumour microenvironment for various cancers. In this study, the authors explore the involvement of cerebrospinal fluid (CSF) cytokines in Group 3 and 4 MB patients with disseminated disease.

**Methods:**

This is an ethics approved, retrospective study of prospectively collected data based at a single institution. Patient clinicpathological data and corresponding bio-materials are collected after informed consent. All CSF samples are interrogated using a proteomic array. Resultant expression data of selected cytokines are correlated with each individual’s clinical information. Statistical analysis is employed to determine the significance of the expression of CSF cytokines in Group 3 and 4 patients with metastatic MB versus non-metastatic MB.

**Results:**

A total of 10 patients are recruited for this study. Median age of the cohort is 6.6 years old. Based on Nanostring gene expression analysis, 5 patients have Group 3 as their molecular subtype and the remaining 5 are Group 4. There are 2 non-metastatic versus 3 metastatic patients within each molecular subtype. Proteomic CSF analysis of all patients for both subtypes show higher expression of CCL2 in the metastatic group versus the non-metastatic group. Within the Group 3 subtype, the MYC-amplified Group 3 MB patients with existing and delayed metastases express higher levels of CXCL1, IL6 and IL8 in their CSF specimens at initial presentation. Furthermore, a longitudinal study of metastatic Group 3 MB observes that selected cytokines are differentially expressed in MYC-amplified metastatic Group 3 MB, in comparison to the non-MYC amplified metastatic Group 3 MB patient.

**Conclusion:**

This study demonstrates higher expression of selected CSF cytokines, in particular CCL2, in metastatic Group 3 and 4 MB patients. Although our results are preliminary, they establish a proof-of-concept basis for continued work in a larger cohort of patients affected by this devastating disease.

## Background

Medulloblastoma (MB) is the most common malignant brain tumour in the paediatric population [1]. MB can be further classified into 4 molecular subtypes (WNT, sonic hedgehog SHH, groups 3 and 4) on the basis of gene expression profiling [2]. Amongst these subtypes, Group 3 and 4 MB have higher propensity to metastasize [3]. In congruency with most human cancers whereby more than 90% of tumour deaths are metastasis-related [4], metastatic MB portends an extremely grim prognosis. This subset of patients usually succumbs to the disseminated spread of MB tumour cells both within and beyond the central nervous system (CNS) [2, 5, 6].

Traditional teaching advocates that MB disseminates to the leptomeninges via primary tumour cell shedding into the cerebrospinal fluid (CSF) followed by distal implantation and growth [7]. However, exact mechanisms of metastasis in MB at this stage, are still incompletely elucidated. The CSF is considered to be a fluid envelope that is responsible for the hydromechanical protection of the CNS. It has an essential role in the homeostasis of the brain parenchymal interstitial fluid and regulation of neuronal functioning [8]. Under physiological circumstances, the continual, directed flow of CSF enables the delivery of various factors within the ventricular system and targeted CNS regions [9]. Separately, inflammation, the 7th hallmark of cancer has been implicated as a key player in tumour development and metastasis [10–12]. In particular, cytokines and their inflammation-related partners are known to coordinate various pro-inflammatory responses within the tumour microenvironment, acting on autocrine and, or paracrine pathways on both malignant and non-malignant cells [13].

As a step towards exploring inflammation in metastatic MB, the authors aim to investigate the involvement of circulating cytokines in the CSF of Group 3 and 4 MB patients with, and without disseminated disease. The 2 main hypotheses for this study are firstly, selected CSF cytokines will be differentially expressed between metastatic Group 3 and 4 MB patients versus non-metastatic Group 3 and 4 patients. Next, there will be different cytokine expressions between metastatic Group 3 and 4 MB, on the basis that these MB subtypes are molecularly distinct from each other.

## Methods

### Study design and patient demographics

This is a single-institution, retrospective study of prospectively collected data, conducted at the KK Women’s and Children’s Hospital. All patients were recruited as part of a hospital ethics board approved study (Singhealth CIRB: 2014/ 2079). Written, informed consent is obtained for study of clinical material collected from both surgery and procedures related to their diagnosis. Inclusion criteria consists of patients who underwent surgery by the Neurosurgical Service, KK Women’s and Children’s Hospital, with histologically proven posterior fossa medulloblastoma tumours, which were further molecularly subtyped to be either Group 3 or Group 4. Patients who do not have medulloblastomas, who have incomplete medical records and, or inadequate clinical material are excluded in this study. For the purposes of this study, margins with regards to extent of resection are adapted from Thompson et al [14]. Here, the extent of resection is based on postoperative imaging and defined as gross total resection (GTR; no residual tumour), near-total resection (NTR; less than 1∙5 cm^2^ remnant tumour), or subtotal resection (STR; more than or equal to 1∙5 cm^2^ remnant tumour) [14].

### Magnetic resonance imaging of the neuro-axis

Imaging details for each patient are obtained from the radiology archives and assessed for completeness. These include a complete magnetic resonance imaging (MRI) of the whole neuroaxis at the time of diagnosis, MRI brain within 48 h post-surgery, and interval MRI of the neuroaxis at scheduled follow-up appointments. All of the study patients have T1-post-gadonilium imaging as part of the sequences. The MRI sequences for the brain and spine are performed on either 1.5 Tesla scanner (GE™ Signa on HD23 platform) or 3 Tesla scanner (Siemens™ Skyra on VE 11A platform) at our institution. Post-contrast T1-weighted images depicting leptomeningeal enhancement and or, areas of contrast-enhancement in distal cranio-spinal areas are included as positive findings for metastasis. In addition, the MRI images also were screened for presence of extraneural lesions away from the primary tumour of interest.

### Histopathology and molecular subtyping examination

Archival glass slides of the resected tumour from each patient are reviewed to confirm the diagnosis of MB. Each tumour is subtyped histologically into classic, large cell/anaplastic and desmoplastic/extensive nodularity categories based on the WHO 2016 classification [15]. Next, RNA is extracted from formalin-fixed and paraffin-embedded tumours and submitted for molecular subtyping (WNT, SHH, Group 3 and Group 4) using a Nanostring™ nCounter assay as previously described [16]. For data analysis, qualitative confidence cut-offs for WNT and SHH subtypes are ≥0.9, and for Group 3 and 4 subtypes are ≥0.7. Results that do not reach these values are classified as ‘indeterminate’.

### Cerebrospinal fluid: collection methods and analysis techniques

Cerebrospinal spinal fluid is collected at time of surgery via a ventricular catheter, or from lumbar puncture during routine surveillance. For cytological examination, samples of CSF are mixed with up to 2 drops of BD Surepath™ preservative fluid (BD system, USA), placed in a cytospin sample chamber and centrifuged at 600 rpm for 6 min. Alcohol-fixed smears (95% ethanol for at least 60 min) are placed in an autostainer machine for Papanicolau staining, and air-dried smears were stained using the Hemacolour® stain. Both cytospin samples and stained smears are then reviewed by our in-house pathologists.

The Human Angiogenesis Antibody Array (Abcam, UK) is a proteome array that targets 43 proteins. (**Supplementary Fig.** **1**). Briefly, CSF samples are centrifuged at 13200 rpm for 5 min prior to experiments, prepared and incubated with a pre-configured antibody array in the form of an immunoblot, as per manufacturer’s instructions. Next, chemiluminescent imaging is performed using the ChemiDoc™ Touch Imaging System version 1.2 (Bio-Rad, USA) and processed via ImageLab version 6.0.1 (Bio-Rad, USA). Membrane blot signal intensities are quantified by ImageJ software (version 1.52a) after normalization to internal controls. Ratios of the respective cytokine expressions and internal control standards are subsequently analysed. All the blots shown in this study are done at 1-min exposure prior to the analysis. This experiment is validated using the Human ProcartaPlex™ Immune Monitoring Panel (ThermoFisher Scientific, USA) for patients who have sufficient remnant CSF specimens. (**Supplementary data A**).

### Statistical analyses

Statistical analyses are performed using 2-tailed Student’s t-test or one-way Analysis of variance (ANOVA). Differences between sample or group means are considered statistically significant when *p*-values < 0.05 (*) or *p*-value < 0.001 (**). Pearson correlation coefficient (Pearson’s *r)* is used to assess strength of association between variables of interest. Graphpad Prism (version 8.4.2) is used to calculate the statistics reported in this study.

## Results

### Patient demographics

A total of 10 patients (6 males and 4 females) are recruited for this study. Median age of the patients is 6.6 ± 3.2 years old (youngest 2 years old and oldest 11 years old). Gross total resection (GTR) is achieved in 6 patients, while the remaining 4 have NTR [14]. Five patients have Group 3 as their molecular subtype and the remaining 5 are Group 4. In our cohort, the 2 patients with a diagnosis of Group 3 molecular subtype, MYC-amplified MB demised rapidly from disease progression. Table 1 shows a summary of the study cohort’s clinicopathological data. Owing to the more clinically aggressive nature of disease in the metastatic Group 3 MB, focus has been given to this subgroup of patients in this study.

**Table 1:** Summary of the study cohort’s clinicopathological information. Note that Patient C* presented without metastasis initially. However, he was found to have bone marrow metastases in bilateral femurs after uneventful completion of his radiotherapy

Patient label	Gender	Extent of resection	Histological subtype	Molecular subtype	MYC Amplification (Yes/ No/ N.D.)	Metastasis at initial diagnosis (radiological)	Location of metastasis	CSF cytology positive (Yes/ No)	Clinical status
A	Female	GTR	Classic	Group 3	No	No	N.A.	No	Disease remission at 15 months
B	Female	GTR	Classic	Group 3	No	No	N.A.	No	Disease remission at 11 months
C*	Male	NTR	Classic	Group 3	Yes	No*	N.A.*	No	Passed away from disease progression
D	Female	NTR	Large cell anaplastic	Group 3	Yes	Yes	Intracranial, spine	No	Passed away from disease progression
E	Female	GTR	Classic	Group 3	No	Yes	Intracranial, spine	No	Alive; receiving RT
F	Male	NTR	Classic	Group 4	N.D.	No	N.A.	No	Disease remission at 20 months
G	Male	GTR	Classic	Group 4	N.D.	No	N.A.	No	Disease remission at 22 months
H	Male	NTR	Classic	Group 4	N.D.	Yes	Intracranial, spine	No	Disease remission at 18 months
I	Male	GTR	Classic	Group 4	N.D.	Yes	Intracranial, spine	No	Passed away at 13 months from relapse
J	Male	NTR	Classic	Group 4	N.D.	Yes	Pituitary gland	No	Disease regression; receiving chemotherapy

### Selective CSF cytokines are differentially expressed between group 3 and 4 MB with and without metastasis

#### There is higher expression of CCL2 in the CSF of patients with metastatic disease versus patient without metastasis for both Group 3 and 4 MB at initial diagnosis

**Fig. 1:**
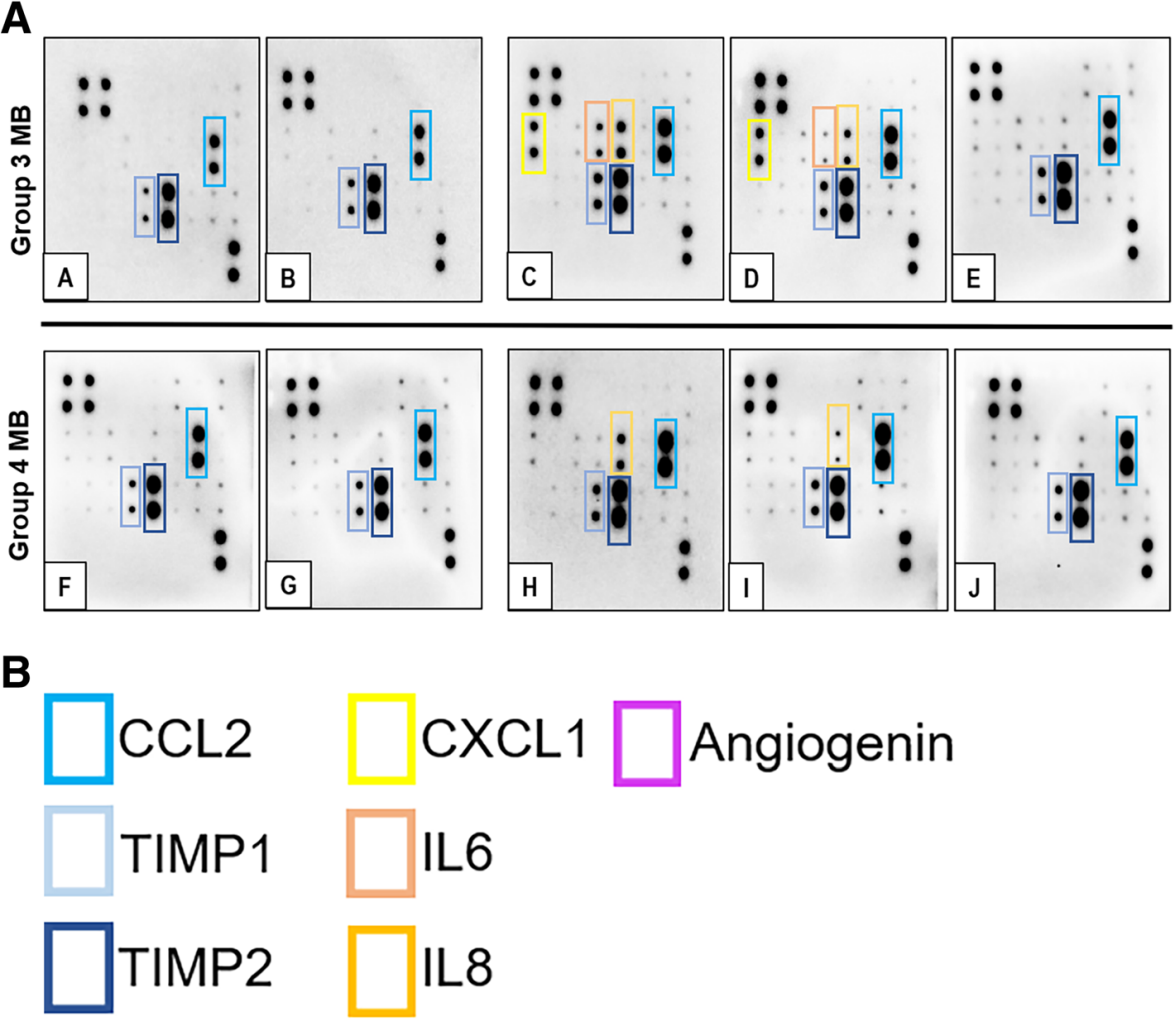
**(A)** Proteome array blot of CSF all patients in study cohort at initial analysis. Patient labels are shown at the bottom left hand corner of each blot. Cytokines of interest are highlighted in coloured outlines and labelled in **(B)**. Full-length blots are presented in **Supplementary Fig. 3**

**Fig. 2:**
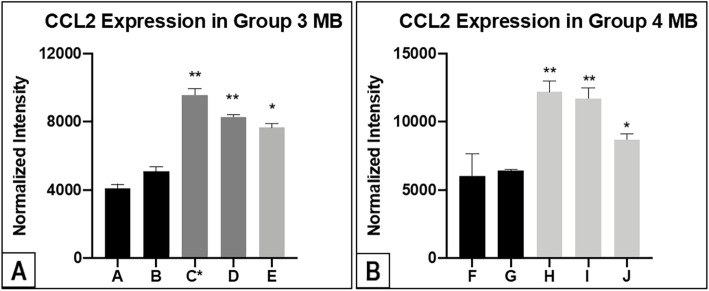
Figures showing intensity quantification results of CCL2 for Group 3 MB **(A)** and Group 4 MB **(B)** from the immunoblot analysis. C* is Patient C who has high expression of CCL2 at the time of initial diagnosis but developed extraneural metastases later

Our results showed that there are comparatively higher expressions of CCL2 in the CSF of patients who had concurrent metastasis at first presentation, regardless of molecular subtype Group 3 or 4 (Figs. 1A, B and 2). Interestingly, the Group 3 MB patient (Patient C) with MYC-amplification who developed interval metastasis later also had a high expression of CCL2 at initial diagnosis. The cytokines, tissue inhibitor of metalloproteinases 1 and 2 (TIMP1 and TIMP2) are also noted to be expressed in all samples. However, further subgroup analysis showed that only TIMP1 had statistically significant association with CCL2 for the metastatic Group 3 MB (Pearson’s *r* 0.912, *p-*value 0.04(*)), in comparison to TIMP2 within the same patients (Pearson’s *r* 0.983, *p-*value 0.09). A similar trend is observed in the validation experiment. (**Supplementary data B**).

#### MYC-amplified group 3 MB patients with existing and delayed metastases express higher levels of CXCL1, IL6 and IL8 in their CSF at initial presentation

**Fig. 3:**
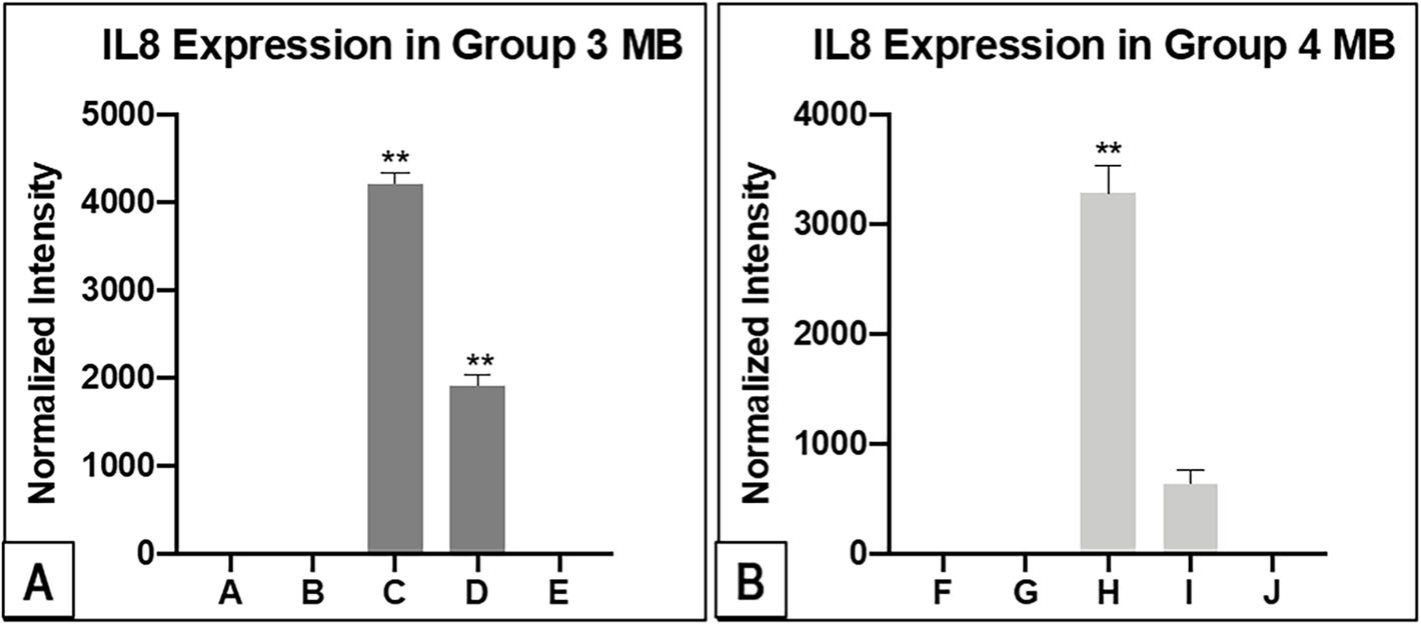
Figures showing intensity quantification results of IL8 for Group 3 MB **(A)** and Group 4 MB **(B)** from the blot analysis. The 2 Group 3, MYC-amplified MB patients have similar trends of high IL8 expression. Conversely, only 1 of the Group 4 metastatic MB has high IL8 expression that is statistically significant

**Fig. 4:**
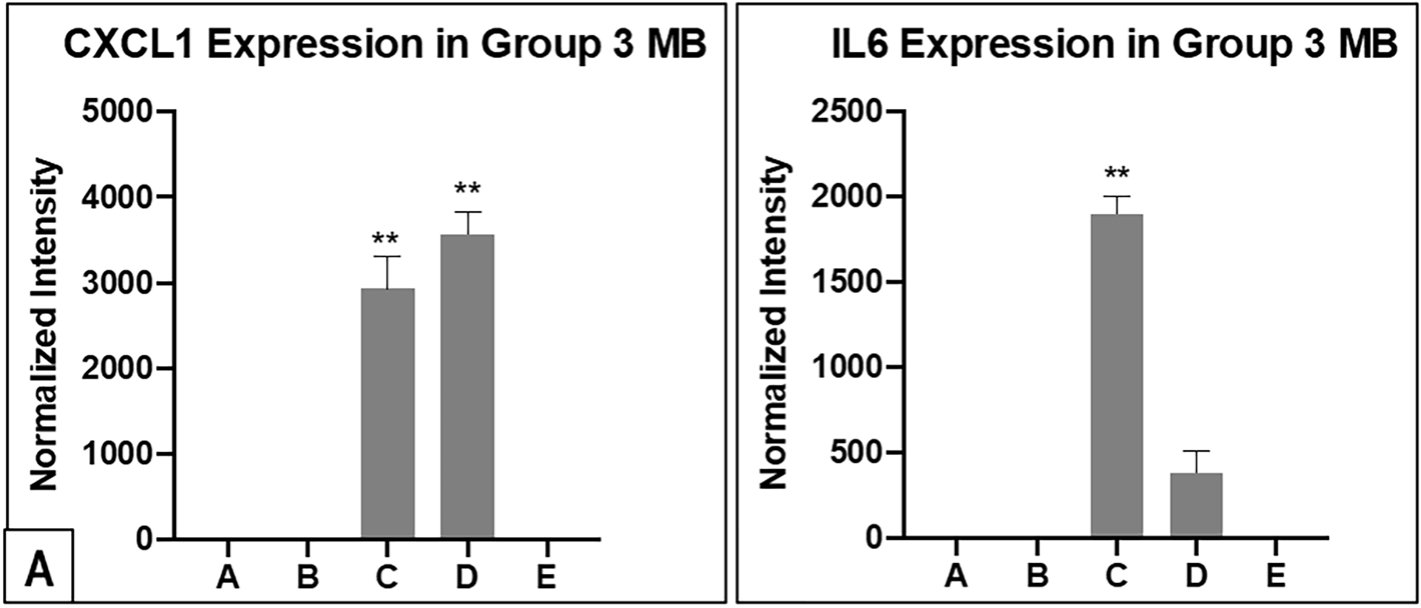
Figures showing intensity quantification results of CXCL1 and IL6 for Group 3 MB from the blot analysis in **(A)** and **(B)** respectively. Similar to the IL8 findings, the 2 Group 3, MYC-amplified MB patients express higher CXCL1 and IL6 in their CSF compared to the rest of Group 3 MB samples. CXCL1 and IL6 do not show sufficient expression in the Group 4 MB cohort after normalization to internal controls

Chemokine (C-X-C motif) ligand 1 (CXCL1), together with interleukin-6 (IL6) and interleukin-8 (IL8) are observed to be significantly higher in the CSF samples of MYC-amplified Group 3 MB with existing and delayed metastases. Only 1 Group 4 patient with metastases expressed statistically significant IL8 in the CSF. (Fig. 3). In addition, CXCL1 and IL6 are not elevated in the CSF of the non-MYC amplified Group 3 MB patient with metastasis and Group 4 MB patients with metastasis. (Fig. 4). Similar trends for CXCL1 and IL6 are observed in the validation experiment. (**Supplementary data B****and Supplementary Fig.** **2**).

### Longitudinal assessment of CSF cytokines in metastatic group 3 MB

**Fig. 5:**
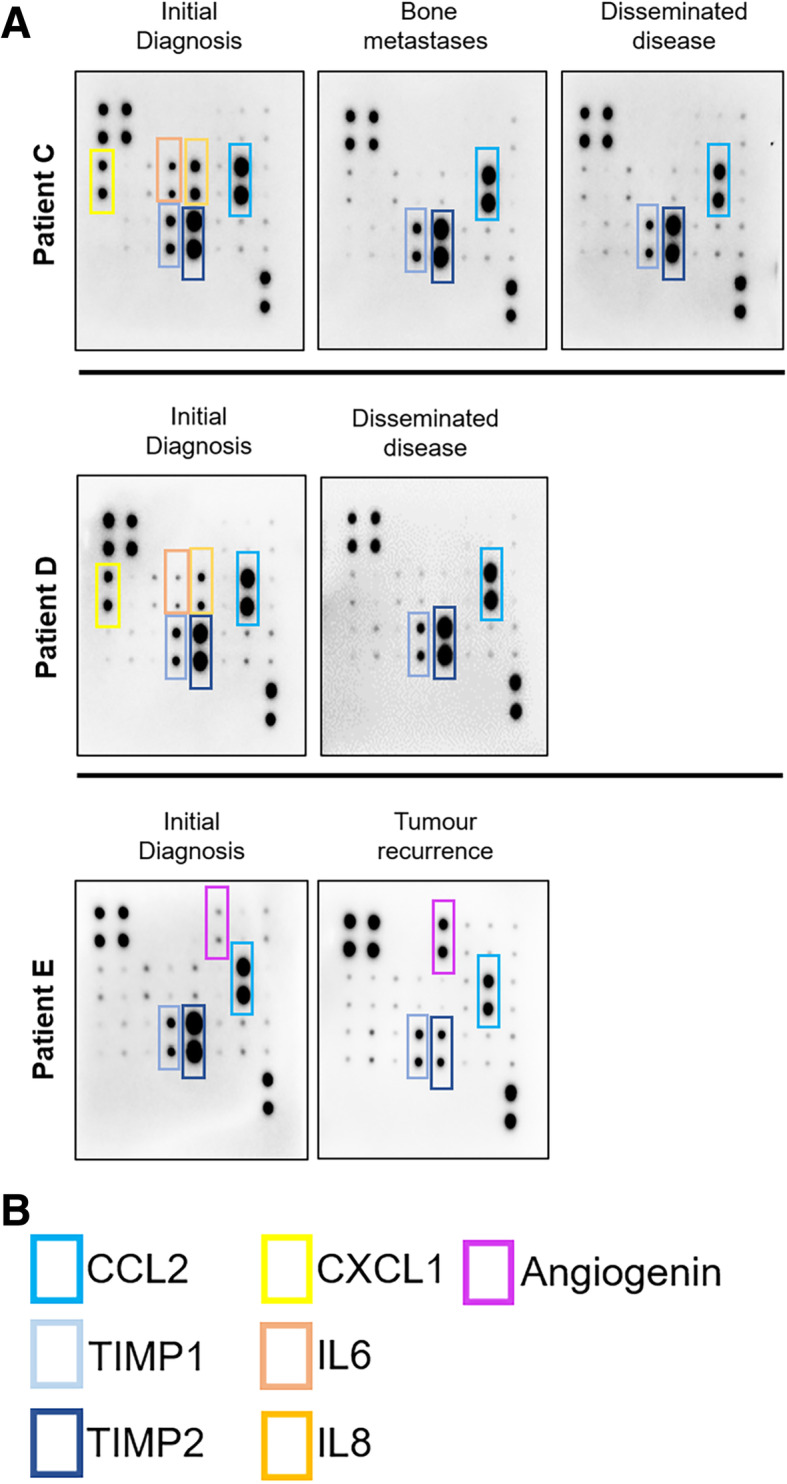
**(A)** Temporal proteome blot depictions of the CSF samples in the Group 3 patients with metastases at various times of their disease. Cytokines of interest are highlighted in coloured outlines and labelled in **(B)**. Full-length blots are presented in Supplementary Fig. 3

Within our study cohort, there are 3 therapeutically challenging patients (Patients C, D and E) within the Group 3 molecular subtype. They present with metastases and early tumour progression. CSF samples collected at various stages of their disease show a steadfast expression of CCL2 in all 3 patients. However, the other cytokines of interest, namely CXCL1, IL6 and IL8 have variable expressions. Notably, there is loss of CXCL1, IL6 and IL8 expression in the CSF for Patients C and D when there was disease progression. Of interest, the cytokine, angiogenin has a significantly higher expression in Patient E (non MYC-amplified) when there is tumour recurrence. (Fig. 5A and B).

#### Clinical case example 1 (Patient C): Group 3 MYC-amplified MB with delayed metastasis

This was a previously well male who presented with headaches. Magnetic resonance imaging reported a posterior fossa lesion associated with obstructive hydrocephalus. There were no other tumours seen on the imaging in the rest of his cranio-spinal axis. He underwent NTR of his tumour. Histology was that of classic MB and molecular subtyping indicated a Group 3 subtype. Fluorescence in situ (FISH) reported MYC-amplification. Cerebrospinal cytology and bone marrow trephine biopsy (BMTB) were negative for malignant cells. The patient commenced adjuvant treatment as per standard risk protocol. He started with radiation therapy (RT) receiving 23 Gy of cranio-spinal irradiation (CSI) and a posterior fossa boost of 54 Gy. Follow-up MRI of the brain and spine performed 30 days after completion of RT showed no evidence of recurrent or new tumour, and CSF cytology remained negative for malignant cells.

One week later, the patient complained of bilateral thigh pain. MRI of both thighs demonstrated marrow lesions. BMTB of his right femur confirmed osseous metastases. Decision was made to continue with chemotherapy. However, surveillance scans performed 1-month after chemotherapy showed tumour recurrence and increase in systemic tumour burden. Approximately 6 weeks later, the patient presented with drowsiness secondary to hydrocephalus. A palliative ventriculoperitoneal shunt (VPS) was inserted. The disease continued to progress rapidly, and the patient eventually succumbed.

#### Clinical case example 2 (Patient D): Group 3 MYC-amplified MB with metastases at presentation

This was a female who presented with seizures associated with a 2-week history of lethargy, nausea and vomiting. Imaging of her brain and spine demonstrated a midline posterior fossa mass with obstructive hydrocephalus. In addition, there was evidence of intracranial metastasis and leptomeningeal disease in the remainder of the MRI brain and spine. She underwent NTR of her posterior fossa tumour which included removal of a left cerebellar metastatic lesion. Intraoperative CSF cytology was negative for malignant cells. Histology reported large cell, anaplastic MB with MYC-amplification via FISH. Molecular subtyping confirmed Group 3 MB. However, on post-operative day 8, she had status epilepticus. Repeat MRI brain scan reported obstructive hydrocephalus with suspicion of tumour progression. An urgent VPS was inserted. CSF collected during shunt surgery, and subsequently during lumbar puncture one week after shunt insertion was positive for tumour cells. BMTB performed concurrently on the day of her lumbar puncture was negative for tumour cells.

The patient was placed on adjuvant treatment for high risk patients. A CSI dose of 36 Gy with posterior fossa tumour bed boost to 55.8 Gy, and spinal metastases boost to 50.4 Gy was given. She was noted to improve clinically during this period. However, approximately 4 weeks post-RT, she presented with fever and abdominal distension. Computed tomographic (CT) scan of her thorax, abdomen and pelvis showed extensive lesions in the pleural and peritoneum cavities that were suspicious for metastases. There was also evidence of intra-abdominal ascities, presumbly from CSF via the VPS catheter. Her VPS valve was tapped to assess the CSF and its cytology did not show tumour cells. Decision was made not to commence chemotherapy. A palliative course was pursued, and the patient passed away within a week of her admission.

#### Clinical case example 3 (Patient E): Group 3 non-MYC amplified MB with metastases at presentation

This female patient was referred to our institution for raised intracranial pressure symptoms secondary to a newly-diagnosed posterior fossa tumour. Her MRI spine also revealed evidence of leptomeningeal disease. She underwent GTR of her tumour uneventfully. Histology confirmed classical MB and subsequent molecular subtyping demonstrated it to be Group 3. The tumour was negative for MYC amplification via FISH. Her CSF was negative for tumour cells. However, prior to commencing RT, the patient was re-admitted for headaches and vomiting. An urgent CT brain scan showed hydrocephalus and evidence of tumour recurrence. She underwent a VPS insertion. Intra-operative CSF collected was once again, negative for malignant cells. At the time of this writing, the patient had completed radiotherapy and was undergoing chemotherapy.

## Discussion

Metastatic medulloblastoma (MB) portends an extremely poor prognosis. The worst outcomes occur in the subset of patients affected by disseminated MB tumour cells both within and beyond the CNS. Extrapolating findings from other human cancers, we are now aware that human body’s response to cancer parallels inflammation, and inflammatory cells and cytokines present in tumours are more likely to contribute to tumour growth and progression [17].

One of these culprit cytokines is CCL2, a pro-inflammatory mediator and chemoattractant. It has a critical role in recruiting macrophages and hence, promoting tumour cell survival [18, 19]. CCL2 is a low molecular weight cytokine with chemoattractant activity. As a prototypic chemokine, CCL2 orchestrates immune cell recruitment to specific sites. It is expressed constitutively for homeostatic functions such as regulating lymphocyte trafficking from blood to lymph nodes. Also, CCL2 is induced during inflammatory responses when leukocytes are required for tissue defence and repair [20]. Recently, Garzia et al. implicates a chemokine signalling axis involving C-C motif ligand 2 (CCL2) and its receptor C-C chemokine receptor type 2 (CCR2) as key players in the haematogenous spread of MB tumour cells [21]. Their pre-clinical study further notes that MYC-amplified Group 3 MB allograft mouse tumours have a higher tendency to express CCL2 [21]. Separately, the MYC gene is a well-characterized oncogene that drives tumorigenesis and metastasis [22], and the mechanisms by which MYC regulates transcription factors involved in metastasis-related processes such as cell migration, and invasion are well documented [22–24]. Despite their findings, we remain uncertain of the mechanisms driving metastases in MB and, CCL2 is not yet clinically demonstrated in patients with metastatic MB at the time of this writing.

At this point, the authors emphasize that the 3 cases of Group 3 metastatic MB are highlighted for the following reasons. Despite their tumours having the same molecular subtype, each of them demonstrates distinct differences in their clinical presentations. Firstly, we observe that higher expression of CCL2 at the time of diagnosis do not reflect the radiological burden of disease in Patient C, versus the rest of the cohort who already have neural metastases at presentation. Furthermore, his CSF cytology was negative. For him, the initial location of disease progression is in the bone marrow while the brain and spine remain radiologically clear of tumour. It is only later that subsequent imaging showed extensive intracranial and leptomeningeal spread. As we are aware, extraneural MB metastases are infrequent [5, 6, 25, 26] and this clinical scenario suggests a haematogenous route, followed by CNS involvement during disease relapse. Here, our study findings suggest that the expression of CCL2 in CSF parallels the existence of metastases (at diagnosis or delayed), and is neither dependent on molecular subtype (that is, Group 3 or 4) nor MYC-amplification (within Group 3 subtypes). In addition, CSF CCL2 expression does not correlate to CSF cytology for our patient cohort.

In corroboration with Garzia et al, our study observes higher CCL2 CSF expression in the metastatic MB patients, especially for the MYC-amplified Group 3 MB. [21]. Putting it all together, our observations potentially justify a role for firstly, further investigation of CCL2 in metastatic MB from a translational perspective; and next, mechanistic exploration of CCL2 in MYC-amplified Group 3 MB, a subset of MB patients with very poor survival despite good surgical cytoreduction and adjuvant treatment.

At the same time, concurrently increased expressions of other cytokines in the CSF results in our study cohort are demonstrated. For example, TIMP1 and TIMP2 expressions, especially the former, are shown to correlate with CCL2 in patients with metastatic Group 3 and 4 MB. Although TIMPs are originally characterized as inhibitors of MMPs, we are now aware that TIMPs have broader biological functions, including effects on cell migration, angiogenesis [27, 28] and cancer progression [29]. The high expression of TIMP1 has also been correlated with adverse prognosis in breast carcinoma [30]. In addition, for the 2 Group 3, MYC-amplified MB patients, interval expression changes in CXCL1 concurrently with IL6 and IL8 are seen. These 3 cytokines have previously been implicated in other tumours as negative prognostic markers [31–34]. Despite these intriguing observations, whether these selected cytokines contribute specifically to the process of MB metastasis needs to be investigated further. With the exception of CCL2, the other cytokines show variable expression at different times of each patient’s disease. The authors postulate that such findings may be a reflection of intertumoral heterogeneity, even within the same molecular subtype.

### Study limitations and future work

The authors acknowledge that there are limitations that should be highlighted. First and foremost, our study population is small. Although our results are novel and potentially may be of translational value, they are preliminary at this stage. Nevertheless, we were able to include CSF findings at initial diagnosis for all patients and longitudinally for a smaller subset of Group 3 MB patients. These findings, in tandem with CCL2 expression in the corresponding tumour tissues, need to be investigated in a larger cohort for translational relevance. Next, our study has mainly focused on the Group 3 metastatic MB subgroup in this paper, as these patients are the most therapeutically challenging. Additional work needs to be done in the remaining MB molecular subgroups to compare their disease trajectories for better disease understanding.

Following that, we are currently working on correlating these cytokines in blood versus CSF in patients with, and without metastatic MB. One of the key reasons is that our cytokines of interest, in particular, CCL2, have been implicated in other diseases [18, 19, 35, 36]. As our study is exploratory at this point, the use of multiplex platforms offers advantages of higher throughput, good sensitivity and lower CSF sample requirement compared to enzyme-linked immunosorbent assay (ELISA) assays. Nonetheless, the development of a focused clinical ELISA kit for CCL2 and its partner cytokines is certainly a consideration in time to come. In addition, targeted, in-depth pre-clinical studies for these cytokines *specifically* in the context of metastatic MB also needs to be established first.

## Conclusion

In summary, we report higher expression of CCL2 in the CSF samples of Group 3 and 4 metastatic MB versus non-metastatic Group 3 and 4 MB in our study cohort. As far as we know, this is the first CSF biomarker to be clinically demonstrated in metastatic MB patients. Furthermore, we show that selected cytokines similarly demonstrate higher expression in the subset of Group 3 MB patients. The authors advocate in-depth studies in a larger population, and international collaborative efforts for better understanding of this devastating disease.

## Supplementary information

**Additional file 1: Supplementary Fig. 1.** Layout of the Human Angiogenesis Antibody Array (Abcam, UK) blot. Although this proteome array targets 43 proteins, expression of the remaining cytokines (not presented in the results) did not show statistical significance or they were not expressed on the array. Note that GRO and MCP-1 are also known as CXCL1 and CCL2 respectively. (https://www.abcam.com/human-angiogenesis-antibody-array-membrane-43-targets-ab193655.html)

**Additional file 2: Supplementary Fig. 2.** Figures showing mean protein expression results of **(A)** CCL2, **(B)** CXCL1, **(C)** IL6 and **(D)** IL8 of non-tumour, Group 3 MB (non-metastatic and metastatic) and Group 4 MB (non-metastatic and metastatic) subtypes. Statistical significance is calculated using 2-tailed Student’s t-test. Abbreviations: N.D. = not detected; METGroup 3 = Group 3 patients with metastases; METGroup 4 = Group 4 patients with metastases

**Additional file 3: Supplementary Fig. 3.** Collated original proteome array blot images of the Human Angiogenesis Antibody Array (Abcam, UK) taken during 1-min exposure using the ChemiDoc™ Touch Imaging System version 1.2 (Bio-Rad, USA) and analysed via ImageLab version 6.0.1 (Bio-Rad, USA). This software uses a .scn file format that is converted to TIFF images for publication. Annotations in the figure show where the blots are cropped and where they are represented in the manuscript figures. Corresponding cytokines in the array are shown in **Supplementary Fig. 1**.

**Additional file 4: Supplementary data A and B. (A)** Materials and methods for the Human ProcartaPlex™ Immune Monitoring Panel (ThermoFisher Scientific, USA). **(B)** Results for CSF interrogation via the Human ProcartaPlex™ Immune Monitoring Panel (ThermoFisher Scientific, USA).

## Data Availability

All data generated or analysed during this study are included in this published article and its supplementary information files.

## Abbreviations

BMTB: bone marrow trephine biopsy
CNS: central nervous system
CSF: cerebrospinal fluid
CSI: craniospinal irradiation
CT: computed tomographic
MB: medulloblastoma
MRI: magnetic resonance imaging
RT: radiation therapy
VPS: ventriculoperitoneal shunt
