# Supplementary figures and images for: Cerebrospinal fluid cytokines in metastatic group 3 and 4 medulloblastoma
Source: BMC Cancer. 2020 Jun 15;20:554. doi: 10.1186/s12885-020-07048-0 (PMC7296667; doi:10.1186/s12885-020-07048-0)

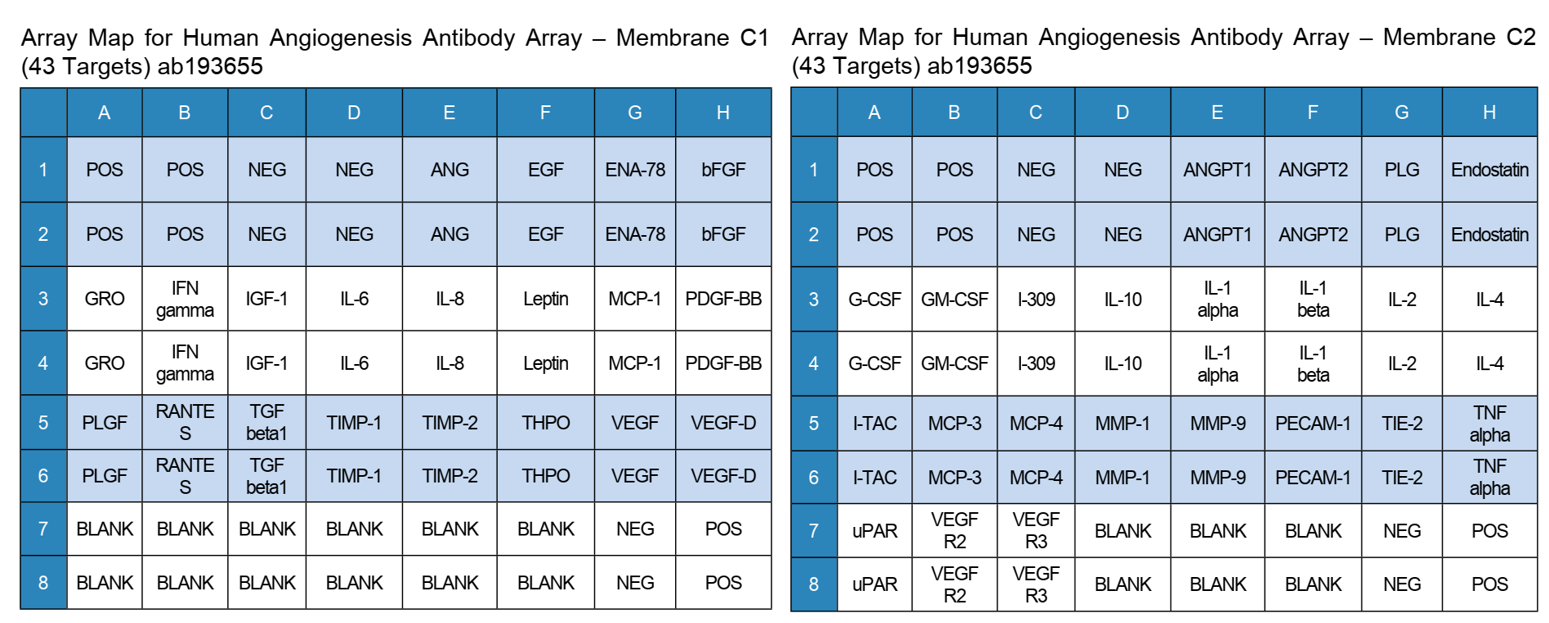

Supplement: Supplementary file 1 — Additional file 1: Supplementary Fig. 1. Layout of the Human Angiogenesis Antibody Array (Abcam, UK) blot. Although this proteome array targets 43 proteins, expression of the remaining cytokines (not presented in the results) did not show statistical significance or they were not expressed on the array. Note that GRO and MCP-1 are also known as CXCL1 and CCL2 respectively. (https://www.abcam.com/human-angiogenesis-antibody-array-membrane-43-targets-ab193655.html) [file 12885_2020_7048_MOESM1_ESM.tif]

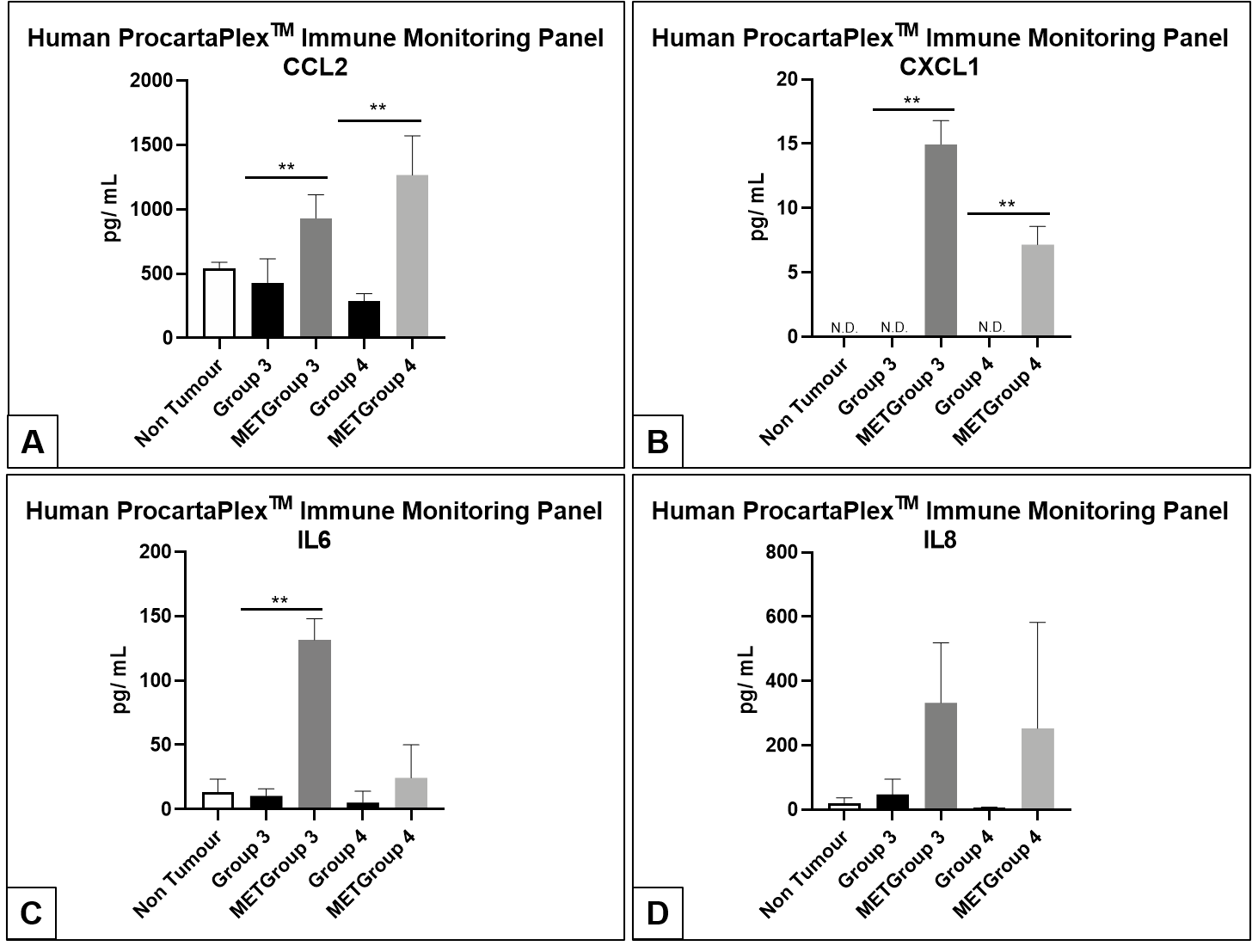

Supplement: Supplementary file 2 — Additional file 2: Supplementary Fig. 2. Figures showing mean protein expression results of (A) CCL2, (B) CXCL1, (C) IL6 and (D) IL8 of non-tumour, Group 3 MB (non-metastatic and metastatic) and Group 4 MB (non-metastatic and metastatic) subtypes. Statistical significance is calculated using 2-tailed Student’s t-test. Abbreviations: N.D. = not detected; METGroup 3 = Group 3 patients with metastases; METGroup 4 = Group 4 patients with metastases [file 12885_2020_7048_MOESM2_ESM.tif]

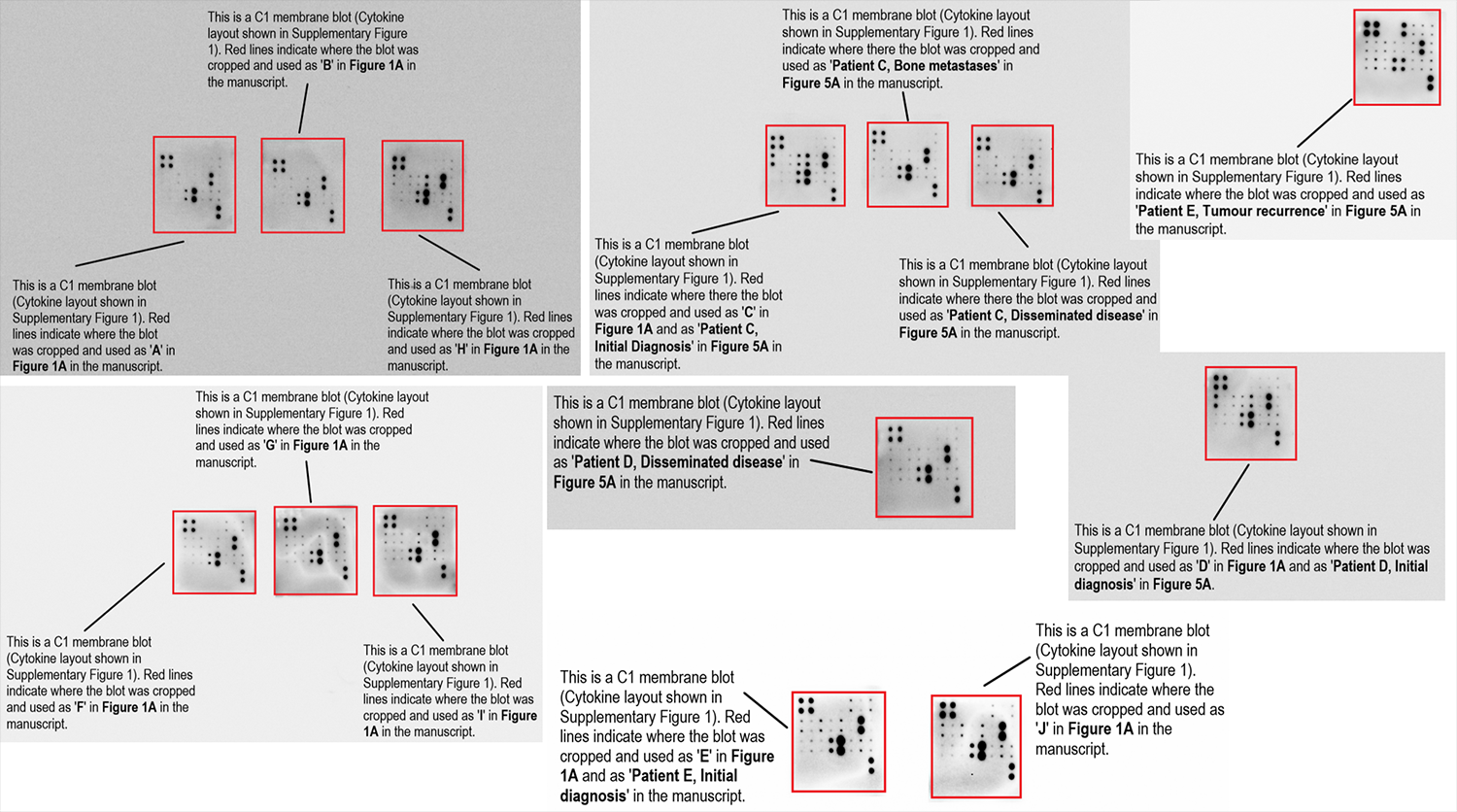

Supplement: Supplementary file 3 — Additional file 3: Supplementary Fig. 3. Collated original proteome array blot images of the Human Angiogenesis Antibody Array (Abcam, UK) taken during 1-min exposure using the ChemiDoc™ Touch Imaging System version 1.2 (Bio-Rad, USA) and analysed via ImageLab version 6.0.1 (Bio-Rad, USA). This software uses a .scn file format that is converted to TIFF images for publication. Annotations in the figure show where the blots are cropped and where they are represented in the manuscript figures. Corresponding cytokines in the array are shown in Supplementary Fig. 1. [file 12885_2020_7048_MOESM3_ESM.tif]
