# Supplementary material for: Cerebrospinal fluid cytokines in metastatic group 3 and 4 medulloblastoma
Source: BMC Cancer. 2020 Jun 15;20:554. doi: 10.1186/s12885-020-07048-0 (PMC7296667; doi:10.1186/s12885-020-07048-0)
Supplement: Supplementary file 4 — Additional file 4: Supplementary data A and B. (A) Materials and methods for the Human ProcartaPlex™ Immune Monitoring Panel (ThermoFisher Scientific, USA). (B) Results for CSF interrogation via the Human ProcartaPlex™ Immune Monitoring Panel (ThermoFisher Scientific, USA). [file 12885_2020_7048_MOESM4_ESM.docx]

**SUPPLEMENTARY DATA (A) and (B)**

1. **Materials and methods**

***Cerebrospinal fluid: collection methods and analysis techniques***

The Human ProcartaPlex™ Immune Monitoring Panel (ThermoFisher Scientific, USA) is a preconfigured multiplex immunoassay kit that measures 65 protein targets: APRIL, BAFF, BLC, CD30, CD40L, ENA-78, Eotaxin, Eotaxin-2, Eotaxin-3, FGF-2, Fractalkine, G-CSF, GM-CSF, GROα (also known as CXCL1), HGF, IFN-α, IFN-ɣ, IL-1α, IL-1β, IL-2, IL-2R, IL-3, IL-4, IL-5, IL-6, IL-7, IL-8, IL-9, IL-10, IL-12p70, IL-13, IL-15, IL-16, IL-17A, IL-18, IL-20, IL-21, IL-22, IL-23, IL-27, IL-31, IP-10, I-TAC, LIF, MCP-1 (also known as CCL2), MCP-2, MCP-3, M-CSF, MDC, MIF, MIG, MIP-1α, MIP-1β, MIP-3α, MMP-1, NGF-β, SCF, SDF-1α, TNF-α, TNF-β, TNF-R2, TRAIL, TSLP, TWEAK, and VEGF-A. Cerebrospinal fluid samples are prepared in duplicates per patient and investigated following the manufacturer’s instructions. Results are read via the Luminex 200 (LX200) system at the end of the experiment.

1. **Results**

Owing to limited availability of clinical material, CSF samples from 8 patients (Patients A, B, C, D, F, G, I and J) were interrogated via the Human ProcartaPlex^TM^ Immune Monitoring Panel (ThermoFisher Scientific, USA). For this experiment, CSF from 2 non-tumour paediatric patients (both are diagnosed with congenital hydrocephalus) are used as controls. The results show similar trends to the proteome array whereby CCL2 and CXCL1 expression levels are significantly higher for the Group 3 and 4 patients with metastases. However, IL6 was only significantly higher for Group 3 metastatic patients, whereas IL8 expression levels did not demonstrate statistical significance for either subtype.
